# A Perception-Based Survey on Practice Patterns Pertaining to the Diagnosis and Management of Allergic Rhinitis in India

**DOI:** 10.7759/cureus.55032

**Published:** 2024-02-27

**Authors:** Vaishali Gupte, Gurmeet Thakur, Ashish Upadhyaya, Subir Jain, Samir Bhargava

**Affiliations:** 1 Medical Affairs, Cipla Ltd, Mumbai, IND; 2 ENT, ENT Centre, Indore, IND; 3 ENT, P.D. Hinduja Hospital, Mumbai, IND; 4 ENT, Hinduhrudaysamrat Balasaheb Thackarey Medical College and Dr. Rustom Narsi Cooper Hospital, Mumbai, IND

**Keywords:** fexofenadine, allergic rhinitis, montelukast, intranasal corticosteroids, antihistamines

## Abstract

Introduction: Allergic rhinitis (AR) is increasingly prevalent in India, affecting a significant portion of the population and adversely impacting their quality of life. This nationwide survey aimed to explore the perceptions and clinical preferences of Indian physicians regarding the perceived prevalence, common symptoms, and various available treatments for AR.

Methods: This cross-sectional, observational, digital questionnaire-based survey was conducted from September 2022 to March 2023, involving physicians sharing insights on prevalence rates, diagnostic approaches, medication preferences, and immunotherapy practices in AR management.

Results: A total of 1608 physicians participated in this survey. The majority of physicians (n=684, 42.5%) reported that the prevalence of AR in routine clinical practice is between 21 and 40%. Physicians also noted a substantial burden of AR with asthma (n=626, around 40%). Total IgE count was reported as a mandatory test for the diagnosis of AR by 47.5% of physicians (n=764). For the management of mild cases of seasonal or perennial AR, 980 (60.9%) physicians preferred fexofenadine as an oral antihistamine of choice. Fluticasone furoate was the preferred intranasal corticosteroid (INCS) option (67.1% of physicians (n=1079)), for the management of patients with moderate to severe AR, the most recommended duration of INCS therapy was two to four months (40.9% of physicians). Doctors recommended a montelukast and antihistamine combination in mild AR (n=152, 9.5%), mild AR not responding to antihistamine alone (n=291, 18.1%), moderate to severe AR along with INCS (n=252, 15.7%), and AR with mild asthma (n=74, 4.6%). The majority of physicians (n=1512, 75.6%) preferred using fexofenadine in combination with montelukast for the management of AR. The majority of physicians (n=839, 52.2%) opined that the efficacy rate of oral montelukast-fexofenadine was 60-90% in the management of mild-moderate AR. Around 55.3% of physicians (n=889) had not used immunotherapy in their clinical practice.

Conclusion: These observations offer a holistic view of how Indian physicians perceive the management of AR, a condition highly prevalent in India and often associated with asthma. It also highlights the treatment strategies employed in their day-to-day clinical practice.

## Introduction

Allergic rhinitis (AR) is a chronic inflammatory respiratory condition resulting from an immune response mediated by IgE antibodies in the nasal mucosa when exposed to allergens. It is often characterized by rhinorrhea, sneezing, itching, and nasal congestion [1-3]. Over the past few years, India has experienced an upsurge in allergic diseases like AR and asthma. Currently, around 22% of Indian adolescents and 9.8% of Indian adults are affected by AR [4]. However, it should be noted that due to the limited availability of comprehensive epidemiological studies in India, especially in rural and suburban regions, these figures may not accurately represent the actual prevalence of this disease [4].

AR has a substantial negative effect on the patient's quality of life (QoL) because it adversely affects sleep quality and cognitive performance [5]. The primary methods for diagnosing AR include the percutaneous skin test and the specific immunoglobulin E (IgE) antibody test for allergens. Diagnostic techniques like nasal provocation testing, nasal cytology (such as the examination of collected secretions, scraping, lavage, and biopsy), nasolaryngoscopy, and intradermal skin testing are seldom used [6]. The majority of patients depend on their physicians for history taking, diagnosis, and treatment of AR; hence, they play a crucial and significant role in the evaluation process as part of the management strategy for AR [7]. The Allergic Rhinitis and Its Impact on Asthma (ARIA) guidelines have provided multiple recommendations, including allergen avoidance, pharmacotherapy, and allergen immunotherapy in the management of AR [8]. Avoiding allergens, utilizing medications that alleviate symptoms, using anti-inflammatory treatments, and considering allergen immunotherapy are the mainstays in the management of AR. In addition to this, oral antihistamines (OAH) are another class of drugs that are routinely used for AR treatment.

The recent advancements in the management of AR include intranasal antihistamines and innovative methods of delivering intranasal steroids [9]. Intranasal corticosteroids (INCS) are widely recognized as a safe and efficient primary treatment for AR, as they are effective in managing AR and can also serve as a preventive measure for perennial AR. In addition to this, they alleviate symptoms like nasal congestion, itching, rhinorrhea, and sneezing that manifest during both the initial and later stages of an allergic reaction [10]. Even though there exist numerous local and global treatment guidelines for the use of INCS, the selection of treatment is often influenced by the physician's perception of the severity of the disease and the patient's preferences regarding medication usage. Despite the presence of these guidelines, many physicians still harbor doubts regarding the advantages and drawbacks associated with the various available treatment options for AR [11-14]. Hence, this survey was conducted to understand the perception of Indian physicians from pan-India sites towards the prevalence of AR, common symptoms of AR observed in Indian patients, and the clinical preferences and utilization of the various available treatments for AR in their clinical practice to enable further improvements.

## Materials and methods

Study design

This was a cross-sectional, observational, digital questionnaire-based survey conducted, which included physicians across India (28 states) for a duration of six months (between September 12, 2022, and March 12, 2023). This was a perception-based survey that exclusively involved the digital participation of ENT surgeons, chest physicians, and consultant physicians responsible for treating AR patients.

Eligibility criteria

Physicians who were treating patients with AR and were willing to participate were included in this study.

Study procedure

The study protocol, physician consent form, and questionnaire were shared digitally with physicians via a link sent over email or phone. Physicians were requested to read the study protocol and the consent form and, after reviewing, physicians who agreed to participate in the study were requested to sign the consent form digitally. Only physicians who provided consent were then requested to participate and respond to all the questions in the questionnaire based on their clinical experience. The participation of physicians in this study was voluntary. The digital questionnaire included a total of 13 questions about the prevalence of AR, common symptoms of AR, and the clinical preferences and utilization of the various available treatments for AR in their clinical practice. Physicians' demographic data, encompassing a range of information such as age, years of practice, practice settings, and location (states) of practice, were also collected in this study. The completed questionnaires were analyzed using descriptive statistical analysis.

Endpoints

The endpoints of this study were to evaluate the perceived prevalence of AR in the clinical practice of physicians in India, to assess the physicians’ choices regarding drugs and formulations for managing mild, moderate, and severe AR symptoms, and to evaluate their perceptions regarding the clinical efficacy and safety of various pharmacotherapies, and the duration of treatment for AR management.

Statistical analysis

The statistical analysis was performed using IBM SPSS Statistics for Windows, Version 27 (Released 2020; IBM Corp., Armonk, New York). Descriptive analysis was used to present the study outcomes. Continuous variables were described as mean and standard deviation (SD), whereas categorical variables were described as numbers and percentages.

## Results

Demographic characteristics of the physicians

A total of 1608 physicians participated in this study (Chest Physician, n=227; Consultant Physician, n=809; ENT surgeons, n=552; others, n=20). The mean (SD) age of the physicians was 44.4±9.9 years, with a mean (SD) practice duration of 15.5±9.6 years. Most of them had private consulting practices (n=1138, 70.8%), followed by those employed at corporate hospitals (n=182, 11.3%), and nursing homes (n=155, 9.6%). The majority of physicians were from the state of Maharashtra (n=188, 11.7%), followed by Uttar Pradesh (n=148, 9.2%), Karnataka (n=126, 7.8%), and West Bengal (n=112, 7.0%) (Table 1).

**Table 1:** Demographic characteristics of the physicians Data presented as n (%), unless otherwise specified. *n=1608, unless otherwise specified. SD, standard deviation.

Parameter	Number of physicians (N=1608)*
Age (years), mean (SD) (N=1602)	44.4 (9.9)
Years of practice, mean (SD) (N=1602)	15.5 (9.6)
Practice setting	
Private consulting practice	1138 (70.8)
Corporate hospital	182 (11.3)
Nursing home	155 (9.6)
Government hospital/academic institute	133 (8.3)
State name	
Maharashtra	188 (11.7)
Uttar Pradesh	148 (9.2)
Karnataka	126 (7.8)
West Bengal	112 (7.0)
Gujarat	107 (6.7)
Andhra Pradesh	107 (6.7)
Haryana	102 (6.3)
Rajasthan	93 (5.8)
Tamil Nadu	90 (5.6)
Telangana	89 (5.5)
Punjab	76 (4.7)
Kerala	76 (4.7)
Madhya Pradesh	66 (4.1)
Delhi	64 (4.0)
Bihar	39 (2.4)
Jammu and Kashmir	36 (2.2)
Uttarakhand	19 (1.2)
Assam	16 (1.0)
Himachal Pradesh	11 (0.7)
Odisha	10 (0.6)
Chhattisgarh	10 (0.6)
Jharkhand	8 (0.5)
Chandigarh	8 (0.5)
Tripura	2 (0.1)
Puducherry	2 (0.1)
Nagaland	1 (0.1)
Meghalaya	1 (0.1)
Arunachal Pradesh	1 (0.1)

Prevalence of AR

The majority of physicians (n=684, 42.5%) reported that the prevalence of AR in routine clinical practice is between 21 and 40%. In subgroup analysis, a similar trend was observed wherein the majority of ENT surgeons (n=255, 46.2%), chest physicians (n=105, 46.3%), and consultant physicians (n=316, 39.1%) reported a prevalence of AR between 21 and 40% (Table 2).

**Table 2:** Prevalence, diagnostic parameters, and comorbidities of AR Data presented as n (%). AR, allergic rhinitis; ENT, ear nose throat; IgE, immunoglobulin E.

Questions	Total (N=1608)	Chest physician (N=227)	ENT (N-=552)	Consultant physician (N=809)	Other (N=14)
What is the prevalence of AR in your routine clinical practice?					
Up to 20%	382 (23.8)	31 (13.7)	92 (16.7)	254 (31.4)	3 (21.4)
21-40%	684 (42.5)	105 (46.3)	255 (46.2)	316 (39.1)	6 (42.9)
41-60%	405 (25.2)	67 (29.5)	147 (26.6)	184 (22.7)	5 (35.7)
61-80%	122 (7.6)	22 (9.7)	51 (9.2)	49 (6.1)	0
>80%	15 (0.9)	2 (0.9)	7 (1.3)	6 (0.7)	0
Which are the tests that are mandatory for the diagnosis of AR?					
Skin prick test	279 (17.4)	35 (15.4)	152 (27.5)	86 (10.6)	3 (21.4)
Total IgE	764 (47.5)	137 (60.4)	227 (41.1)	392 (48.5)	5 (35.7)
Allergen-specific IgE blood test	565 (35.1)	55 (24.2)	173 (31.3)	331 (40.9)	6 (42.9)
What is the percentage of your patients with AR who also have asthma?					
Up to 20%	566 (35.2)	29 (12.8)	267 (48.4)	263 (32.5)	5 (35.7)
21-40%	626 (38.9)	92 (40.5)	193 (35)	335 (41.4)	5 (35.7)
41-60%	318 (19.8)	79 (34.8)	75 (13.6)	160 (19.8)	1 (7.1)
61-80%	76 (4.7)	24 (10.6)	12 (2.2)	38 (4.7)	2 (14.3)
>80%	22 (1.4)	3 (1.3)	5 (0.9)	13 (1.6)	1 (7.1)

Diagnostic parameters in AR

According to 764 (47.5%) physicians, the measurement of total IgE count is a mandatory test for diagnosis of AR, whereas the remaining 35.1% and 17.4% of physicians (n=565 and 279) considered allergen-specific IgE blood test and skin prick test, respectively, as mandatory tests for the diagnosis of AR (Table 2).

Comorbidities

A total of 38.9% (n=626) and 35.2% (n=566) of physicians reported that 21-40% and up to 20%, respectively, of their patients with AR have asthma. The majority of chest physicians (92 (40.5%) and 79 (34.8%)) opined that 21-40% and 41-60% of their patients with AR have asthma, whereas the majority of ENT surgeons (267 (48.4%) and 193 (35%)) and consultant physicians (263 (32.5%) and 335 (41.4%)) opined that up to 20% and 21-40% of patients with AR have asthma respectively (Table 2). 

Medication preferences for AR management

For the management of mild cases of seasonal or perennial AR, the majority of physicians (n=980, 60.9%) preferred fexofenadine as the oral antihistamine of choice. Bilastine and levocetirizine were also recommended by 16.8% (n=270) and 18.8% (n=303) of physicians, respectively. For managing moderate to severe AR symptoms, the majority of physicians (1079 (67.1%)), selected fluticasone furoate as the preferred INCS option, while 24.3% (n=391) opted for fluticasone propionate. When assessing the clinical outcomes, approximately half of the physicians (802 (49.9%)) reported better results with fluticasone furoate, 262 (16.3%) reported better results with fluticasone propionate, and 27.2% of physicians (n=438) opined that they haven’t noticed any major differences between the two molecules and have got good results with both the molecules (Table 3).

**Table 3:** Medication preferences for AR management Data presented as n (%). AR, allergic rhinitis; ENT, ear nose throat; INCS, intranasal corticosteroid; SCIT, subcutaneous immunotherapy; SLIT, sublingual immunotherapy.

Questions	Total (N=1608)	Chest physician (N=227)	ENT (N-=552)	Consultant physician (N=809)	Other (N=14)
Among the following oral antihistamines, which ones do you prefer in the management of mild cases of seasonal or perennial AR?					
Fexofenadine	980 (60.9)	126 (55.5)	340 (61.6)	504 (62.3)	5 (35.7)
Levocetirizine	303 (18.8)	48 (21.1)	91 (16.5)	158 (19.5)	5 (35.7)
Bilastine	270 (16.8)	40 (17.6)	104 (18.8)	123 (15.2)	3 (21.4)
Desloratadine	32 (2.0)	7 (3.1)	10 (1.8)	15 (1.9)	0
Cetirizine	23 (1.4)	6 (2.6)	7 (1.3)	9 (1.1)	1 (7.1)
Which is your preferred INCS for the management of the symptoms of moderate to severe AR?					
Fluticasone furoate	1079 (67.1)	150 (66.1)	433 (78.4)	488 (60.3)	7 (50)
Fluticasone propionate	391 (24.3)	57 (25.1)	90 (16.3)	236 (29.2)	5 (35.7)
Mometasone furoate	89 (5.5)	12 (5.3)	26 (4.7)	49 (6.1)	1 (7.1)
Budesonide	49 (3.0)	8 (3.5)	3 (0.5)	36 (4.4)	1 (7.1)
Have you ever noticed any differences in clinical outcomes between fluticasone furoate and fluticasone propionate?					
Yes, clinical outcomes are better with fluticasone furoate	802 (49.9)	119 (52.4)	317 (57.4)	360 (44.5)	3 (21.4)
Yes, clinical outcomes are better with fluticasone propionate	262 (16.3)	37 (16.3)	61 (11.1)	163 (20.1)	1 (7.1)
I haven’t noticed any major differences between the two molecules and have got good results with both	438 (27.2)	58 (25.6)	153 (27.7)	217 (26.8)	7 (50)
I have been using only one molecule and have no experience with the other one	97 (6.0)	12 (5.3)	18 (3.3)	65 (8.0)	2 (14.3)
Any other outcome, please specify	9 (0.6)	1 (0.4)	3 (0.5)	4 (0.5)	1 (7.1)
Which immunotherapy do you prefer?					
I don't prescribe immunotherapy	889 (55.3)	103 (45.4)	277 (50.2)	500 (61.8)	6 (42.9)
SCIT	339 (21.1)	56 (24.7)	96 (17.4)	183 (22.6)	4 (28.6)
SLIT	380 (23.6)	68 (30)	179 (32.4)	126 (15.6)	4 (28.6)

Immunotherapy preferences

Around 55.3% of physicians (n=889) stated that they do not prescribe immunotherapy, while 23.6% (n=380) preferred sublingual immunotherapy (SLIT), and 21.1% (n=339) preferred subcutaneous immunotherapy (SCIT) (Table 3).

Combination therapies for AR

The majority of physicians (829 (51.6%)) reported that they recommend a combination of montelukast and antihistamine in patients with all listed conditions (seasonal/perennial AR with mild symptoms; seasonal/perennial AR with mild symptoms not responding to antihistamine alone; seasonal/perennial AR with moderate-to-severe symptoms, along with INCS; and seasonal/perennial AR with mild asthma). Specifically, 18.1% (n=291) recommended it for seasonal/perennial AR with mild symptoms not responding to antihistamine alone, and 15.7% (n=252) advised this combination for seasonal/perennial AR with moderate-to-severe symptoms, alongside INCS. The majority of physicians (1215 (75.6%)) preferred using fexofenadine in combination with montelukast for the management of AR. A total of 47.1% (n=757) of the physicians considered using azelastine and fluticasone in patients not responding to INCS alone, patients with seasonal or perennial AR with moderate to severe symptoms, and patients who need rapid symptom relief (Table 4).

**Table 4:** Combination therapies for AR Data presented as n (%). AR, allergic rhinitis; ENT, ear nose throat; INCS, intranasal corticosteroid.

Questions	Total (N=1608)	Chest physician (N=227)	ENT (N=552)	Consultant physician (N=809)	Other (N=14)
When do you recommend the combination of montelukast and antihistamine?					
Seasonal/perennial AR with mild symptoms	152 (9.5)	22 (9.7)	55 (10)	75 (9.3)	0
Seasonal/perennial AR with mild symptoms not responding to antihistamine alone	291 (18.1)	35 (15.4)	88 (15.9)	162 (20)	6 (42.9)
Seasonal/perennial AR with moderate-to-severe symptoms, along with INCS	252 (15.7)	42 (18.5)	105 (19)	101 (12.5)	3 (21.4)
Seasonal/perennial AR with mild asthma	74 (4.6)	14 (6.2)	27 (4.9)	32 (4)	1 (7.1)
All of the above	829 (51.6)	111 (48.9)	272 (49.3)	437 (54)	4 (28.6)
I don't use this combination	10 (0.6)	3 (1.3)	5 (0.9)	2 (0.2)	0
Which drug do you prefer in combination with montelukast for the treatment of AR?					
Fexofenadine	1215 (75.6)	165 (72.7)	410 (74.3)	626 (77.4)	9 (64.3)
Cetirizine	31 (1.9)	6 (2.6)	5 (0.9)	18 (2.2)	1 (7.1)
Levocetirizine	439 (27.3)	66 (29.1)	155 (28.1)	213 (26.3)	2 (14.3)
Bilastine	466 (29.0)	72 (31.7)	172 (31.2)	216 (26.7)	4 (28.6)
I do not use combinations	7 (0.4)	2 (0.9)	3 (0.5)	1 (0.1)	1 (7.1)
In which patient profile do you recommend the combination of azelastine and fluticasone?					
Patient not responding to INCS alone	235 (14.6)	22 (9.7)	78 (14.1)	130 (16.1)	4 (28.6)
Seasonal / perennial AR with moderate to severe symptoms	154 (9.6)	21 (9.3)	49 (8.9)	81 (10)	3 (21.4)
Patients who need rapid symptom relief	343 (21.3)	47 (20.7)	142 (25.7)	153 (18.9)	1 (7.1)
All the above	757 (47.1)	129 (56.8)	212 (38.4)	407 (50.3)	5 (35.7)
I don't use this combination	119 (7.4)	8 (3.5)	71 (12.9)	38 (4.7)	1 (7.1)

Duration of therapies in patients with AR

**Figure 1:**
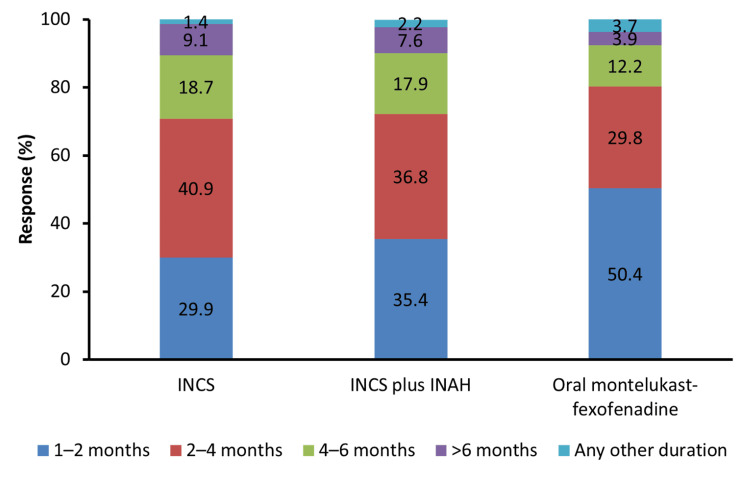
Recommended duration of therapy with different molecules INCS, intranasal corticosteroid; INAH, intranasal H1 antihistamine.

**Figure 2:**
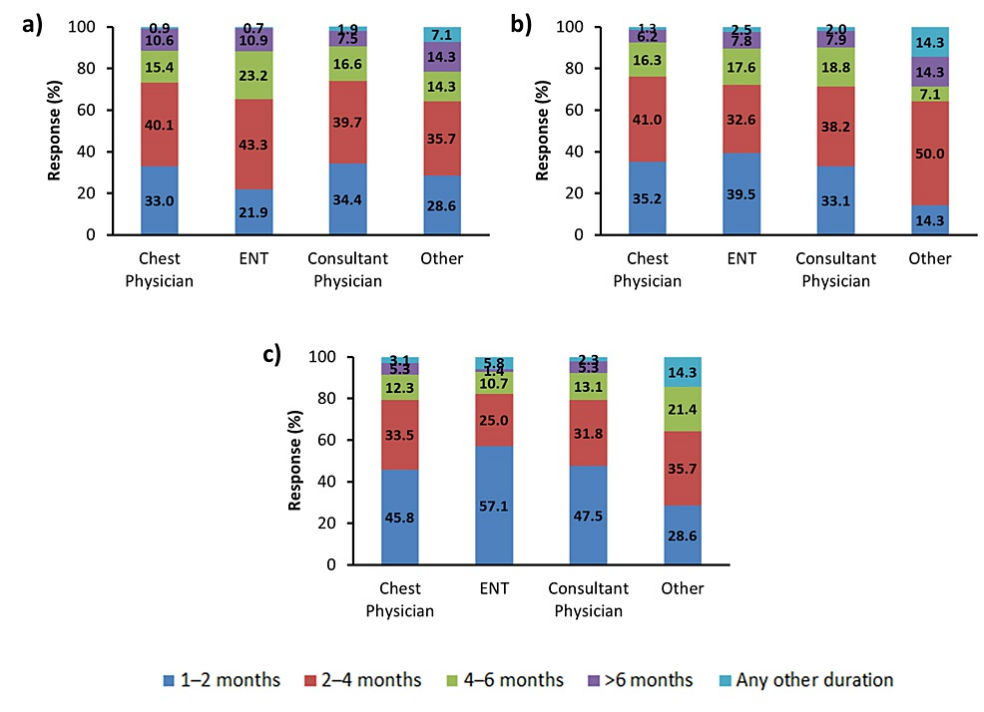
Recommended duration of therapy with (a) INCS molecules (b) INCS plus INAH molecules, and (c) oral montelukast: fexofenadine molecules for patients with moderate to severe AR ENT, ear nose throat; INCS, intranasal corticosteroid; INAH, intranasal H1 antihistamine.

For the management of patients with moderate to severe AR, the most recommended duration of INCS therapy was 2-4 months, as suggested by 40.9% of the physicians. The combination of INCS and INAH was recommended for 2-4 months by 36.8% of physicians and for 1-2 months by 35.4% of physicians. The most recommended duration of treatment with oral montelukast-fexofenadine for the management of moderate to severe AR was 1-2 months (50.4% of physicians) (Figure 1). In the subgroup analysis, which included different specialties such as ENT surgeons, chest physicians, and consultant physicians, it was observed that the most recommended duration of INCS therapy was 2-4 months (chest physicians, 40.1%; ENT surgeons, 43.3%; and consultant physicians, 39.7%). When considering the combination of INCS and INAH therapy, the majority of chest physicians (41.0%) and consultant physicians (38.2%) recommended 2-4 months of combination therapy, whereas ENT surgeons (39.5%) suggested a 1-2 months duration for the INCS and INAH combination therapy. For oral montelukast-fexofenadine therapy, 1-2 months was the most recommended duration of therapy by chest physicians (45.8%), ENT surgeons (57.1%), and consultant physicians (47.5%) (Figure 2A-C). The above data showed the difference in opinion of ENT surgeons, chest physicians, and consultant physicians regarding the optimal duration of the combination of INCS and INAH therapy for the management of AR.

Efficacy and safety

The majority of physicians (839 (52.2%)) opined that the efficacy rate of oral montelukast-fexofenadine was 60-90% in the management of mild to moderate AR, and 14.8% (n=238) of physicians reported the efficacy rate exceeding 90%. Regarding safety, a majority of physicians (1241 (77.2%)) reported that INCS is safe and well-tolerated, with no safety concerns. However, 22.8% (n=367) of physicians reported safety concerns, primarily related to nasal atrophy and intranasal crusting (Table 5).

**Table 5:** Efficacy and safety of drugs in AR Data presented as n (%). AR, allergic rhinitis; ENT, ear nose throat; INCS, intranasal corticosteroid.

Questions	Total (N=1608)	Chest physician (N=227)	ENT (N-=552)	Consultant physician (N=809)	Other (N=14)
How do you rate the efficacy rates (% respondent) of the Oral montelukast-fexofenadine in the management of moderate-to-severe AR?					
10-30%	89 (5.5)	14 (6.2)	20 (3.6)	55 (6.8)	0
30-60%	442 (27.5)	86 (37.9)	137 (24.8)	213 (26.3)	4 (28.6)
60-90%	839 (52.2)	102 (44.9)	302 (54.7)	424 (52.4)	9 (64.3)
>90%	238 (14.8)	25 (11)	93 (16.8)	117 (14.5)	1 (7.1)
How would you rate the safety of INCS in the long-term management of the symptoms of AR?					
Is safe and well tolerated, with no safety concerns	1241 (77.2)	187 (82.4)	448 (81.2)	595 (73.5)	8 (57.1)
Some safety concerns observed in terms of nasal atrophy and intranasal crusting	367 (22.8)	40 (17.6)	104 (18.8)	214 (26.5)	6 (42.9)

## Discussion

This PAN-India study provides a comprehensive picture of Indian physicians’ perception with respect to the management of AR and aids in better understanding the treatment strategies that are currently used in their routine clinical practice. According to the perceptions of the majority of physicians, this study revealed that the perceived prevalence of AR in clinical practice falls within the range of 21-40%. The available clinical evidence indicates that the prevalence of AR has been on the rise in India. The reported incidence of AR in India falls within the range of 20-30% [2]. A survey-based study from India reported that the majority of physicians (63.0%) mentioned that the prevalence of AR falls within the range of 10-30% [15]. 

The presence of AR alongside asthma results in the aggravation of asthma symptoms and increased susceptibility to hospitalizations for asthma when compared to individuals with asthma alone [16,17]. In this study, the prevalence rate of asthma in patients with AR was in the range of 21-40%, as suggested by 38.9% of physicians. This was similar to the Asia-Pacific survey of physicians on asthma and AR, which inferred a 28% prevalence rate of asthma in patients with AR [18]. A study by Demoly et al. also observed that among the patients with AR, around 14.1% of patients had asthma [19]. 

In the present study, the majority of physicians (60.9%) preferred fexofenadine as the oral antihistamine of choice for the management of mild cases of seasonal or perennial AR, and more than half of the participating physicians (51.6%) preferred a combination of montelukast and antihistamine for management of AR in different clinical scenarios including mild to moderate to severe symptoms, who are not responding to antihistamine alone or those with mild asthma. Additionally, a small proportion (15.7%) of respondents recommended this combination therapy for cases of seasonal or perennial AR with moderate-to-severe symptoms, in conjunction with INCS. These results are in concordance with the recommendations and guidelines from the American Academy of Otolaryngology, Head and Neck Surgery, Allergic Rhinitis and its Impact on Asthma, which states that physicians are advised to recommend oral second-generation/less sedating antihistamines for patients whose primary complaints related to AR are sneezing and itching. For patients with seasonal, perennial, or episodic AR, physicians may consider offering intranasal antihistamines. However, oral leukotriene receptor antagonists (LTRAs) should not be recommended as the primary therapy for AR patients [12]. Another survey-based study also observed that 52.8% of physicians preferred using oral H1 antihistamines as their initial choice of treatment. Following that, 25.6% of physicians favored a combination of oral H1 antihistamines and LTRA. Among the oral combination therapies, most physicians showed a preference for oral H1 antihistamines with LTRA. This was followed by a tendency to opt for a combination of oral H1 antihistamines with decongestants and a combination of oral H1 antihistamines with INCS [15]. Furthermore, a study by Prabhat et al. also reported similar results; around 41% of physicians opted for oral antihistamines as the first drug of choice [5].

In this study, the majority of physicians (67.1%), selected fluticasone furoate as the preferred INCS option, while 24.3% opted for fluticasone propionate for managing moderate to severe AR symptoms. This can be attributed to the notable efficacy of fluticasone furoate, a novel topical INCS, in alleviating both nasal and ocular symptoms associated with AR, with commendably low occurrence of adverse side effects [20]. A cross-sectional, prospective, pan-India survey of 300 physicians reported parallel observations with fluticasone (55%) being the first choice of INCS among Indian physicians [5]. Similarly, observations from a study by Yanez et al. corroborate with the opinions of physicians from the present study, wherein authors witnessed that a significant majority of patients opted for fluticasone furoate nasal sprays (52%) over mometasone furoate nasal sprays (32%) for AR (p<0.001) [21]. Hence, the present study along with previous studies indicates that both the patient's and the physician's viewpoints converged on choosing fluticasone furoate as the preferred INCS for treating AR.

The combination therapy of montelukast with an antihistamine offers enhanced and complementary effects, effectively reducing symptoms of AR [22]. The physicians in this study held positive perceptions regarding the efficacy of oral montelukast-fexofenadine. Approximately 52% of them reported efficacy rates ranging from 60% to 90% in managing mild to moderate AR, and around 15% of physicians reported that this combination therapy exceeded an efficacy rate of 90%. These data align with observations from a randomized double-blind clinical trial that conducted a comparative analysis of two combinations, montelukast-levocetirizine and montelukast-fexofenadine, and revealed that the mean change in the total nasal symptom score (TNSS) from baseline to 4 weeks was higher in the montelukast-fexofenadine group when compared to montelukast-levocetirizine group (9.4 vs 8.0; P=0.0033) [22].

This study results observed that the majority of physicians (55.3%) do not prescribe any immunotherapy, whereas the remaining proportion of physicians preferred immunotherapy either in the form of SLIT or SCIT. The physicians who chose to refrain from recommending it to patients with AR may have limited knowledge about immunotherapy. This was similar to the results observed in a prospective observational study, which concluded that primary care physicians (69.5%) appeared to lack knowledge and awareness regarding the advantages of recent antihistamine medications and treatments, such as immunotherapy [7]. In addition to this, the clinical practice guidelines on AR states that the physicians should provide or make a referral to a healthcare provider capable of offering immunotherapy (either sublingual or subcutaneous) for patients with AR who do not experience sufficient relief from symptoms with pharmacologic therapy, whether used alone or in combination with environmental controls [12]. Hence, the present study along with previous studies constantly highlights limited awareness and knowledge about optimal usage conditions for immunotherapy during the management of AR.

AR remains a significant global health concern due to its escalating prevalence. The pivotal role of physicians in AR management cannot be understated, yet their awareness and understanding of various aspects of AR are hindered by the absence of standardized aids. The data highlight a rising trend of AR in clinical practice from physicians across India. It falls within the range of 20-30%, with physicians acknowledging the burden it poses [2,23]. As we strive to address the growing impact of AR, further research and education among physicians are essential steps toward better patient care and outcomes.

This study has several limitations. This cross-sectional survey relied on the data collected from physicians' recall, such as the frequency of patients treated for AR and the percentage of their practice devoted to this treatment. This introduces the possibility of recall bias. The participating chest physicians, consultant physicians, and ENT surgeons were predominantly from urban areas. As a result, the findings of this study may not be representative of rural areas in the country. Future research should consider conducting survey-based studies in different settings, including physicians from urban as well as rural hospitals.

## Conclusions

This nationwide survey explored the perception of physicians across India about the management of patients with AR and the overall responses of physicians provide key insights into the various aspects of AR management. AR continues to be a burgeoning concern in India, with a high perceived prevalence in clinical practice ranging from 21% to 40% and the majority of physicians noted the perceived burden of AR with asthma to be 40%. There was a variation noted in the practice patterns of different physicians; however, the majority of physicians prefer oral montelukast in combination with fexofenadine for a period of 1-2 months. Fexofenadine was a preferred choice among physicians, considering that it was observed to be efficient and well-tolerated in patients as stated by the majority of physicians across India. The use of immunotherapy for the management of AR was not used by the majority of physicians, which emphasizes the need for education and awareness of the therapy. 
